# Remote Control of Gold–Iron Nanowires Using Low-Frequency 1 Hz Magneto-Mechanical Therapy and Cesium 137 0.662 MeV Radiotherapy for Treatment of Glioblastoma Multiforme

**DOI:** 10.1021/acsami.5c05004

**Published:** 2025-05-28

**Authors:** Jonathan Taylor, George Greaves, Chris Clement Phillips, Matthew Williams, Mary P. Ryan, Alexandra E. Porter

**Affiliations:** † Department of Materials and London Centre for Nanotechnology, Imperial College, London SW7 2AZ, U.K.; ‡ Department of Physics, Imperial College London, London SW72AZ, U.K.; § Imperial College Healthcare NHS Trust, Charing Cross Hospital, Fulham Palace Rd, London W6 8RF, U.K.

**Keywords:** glioblastoma multiforme, magneto-mechanical therapy, radiotherapy, gold−iron nanowire, electrochemical deposition

## Abstract

Glioblastoma (GBM) is an extremely infiltrative brain cancer that is impossible to fully remove surgically and almost always recurs at the borders of the resection cavity. There is increasing focus on inducing cancer cell death using magneto-mechanical therapy (MMT), which involves energy conversion of an external low-frequency magnetic field into mechanical forces using magnetic nanoparticles. Here, we combined MMT with enhanced radiotherapy (RT)—the standard of care treatment for GBM—to increase the efficiency of treatment using gold–iron nanowires (AuFe NWs). The magnetic iron component of the nanowires mechanically rotates, inducing cellular damage, and the gold scatters X-rays due to its high atomic number, enhancing the local RT dose. We show that reproducible synthesis of AuFe NWs with different ratios of gold:iron can be achieved using a hard-template electrochemical method, controlling composition by tuning the deposition current. Ratios with best-performing iron percentages were selected for computational modeling to predict which frequency should be applied *in vitro* on a GBM cell line. *In vitro* testing, using a cell metabolism assay, and the optimal frequency and gold:iron ratio, demonstrated that applying MMT alongside RT resulted in a synergistic effect, reducing cell viability significantly by ∼60% (as compared with a 30% reduction for RT, with/without AuFe NWs), and a 20% reduction for MMT (with AuFe NWs). The increased efficacy of RT, post-MMT, was attributed to the higher association of the nanowires with the cells following application of the magnetic field and local membrane damage.

## Graphical abstract

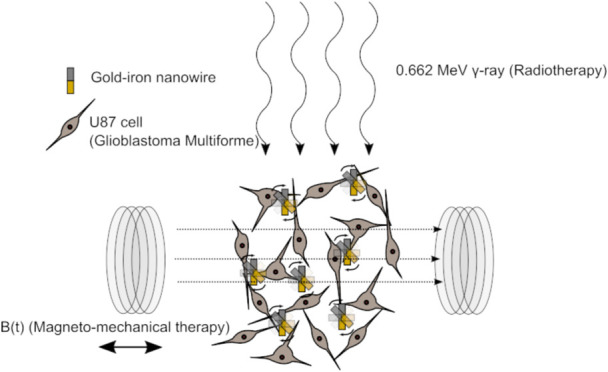

## Introduction

Glioblastoma (GBM) is a malignant primary brain tumor with very poor prognosis, with a median survival time following diagnosis of around 15 months that can develop very rapidly and is difficult to treat with surgery, RT, or chemotherapy.^1^ Conventional therapies such as chemotherapy and RT have improved survival rates for some types of brain cancer. However, malignant primary brain tumors such as GBM still have poor prognosis and are difficult to treat with surgery, RT, or chemotherapy. Surgery to remove all or part of the tumor may be possible, depending on the location of the tumor in the brain. RT uses high-energy ionizing radiation to damage the cancerous cells but can cause severe side effects from localized tissue damage due to radiation. Chemotherapy can be used for treatment with RT and surgery. Standard of care uses the drug Temozolomide as an adjuvant; however, this only extends life by 15–16 months and has deleterious side effects such as headaches and nausea. Another approach is to deliver drugs into a biodegradable wafer for local administration following surgery.^2^ New treatment strategies are vitally important to improve quality of life and extend overall survival of patients with the disease.^1^

In recent years, novel therapies like tumor-treating electric fields^3,4^ and magneto-thermal therapy^5^ have shown promising results in clinical trials, extending overall survival further by a few months. Since RT requires a careful balance between enhancing damage to the tumor and reducing harm to the patient, localizing and reducing the dose is a promising approach that should in future improve its success. High-atomic-number (*Z* number) materials such as gold and hafnium dioxide scatter incident radiation and can be administered within the tumor and locally enhance the radiation dose.^6^ For example, hafnium dioxide nanoparticle radiosensitizers are in phase 2/3 clinical trials to treat advanced soft-tissue sarcoma.^7^ In the randomized trial, patients that received hafnium dioxide nanoparticles intratumorally with preoperative external-beam RT (50 Gy in 25 fractions) followed by surgery showed a 2× greater response post-surgery than those receiving RT alone with surgery.^7^ Gold nanoparticles have also been the focus of many radiosensitization studies and successfully sensitize tissue at higher energies than predicted from physical scattering (*e.g*., ref 6 and reviewed in refs 8,9 ). Iron oxide nanoparticles can also radiosensitize cancer cells *in vitro* at MV energies and thus have the potential to be developed into radiosensitization and imaging tools.

Magnetic nanoparticle-mediated magneto-thermal therapies destroy tumors by causing hyperthermia to the cancerous cells.^5,11^ Magneto-mechanical therapy (MMT) (reviewed in ref 12) is to apply an external low-frequency magnetic field to the tissue to rotate the magnetic nanoparticles causing mechanically induced membrane damage to the cancer cells (*e.g*., refs 12–16 ). MMT could provide a new treatment modality for inhibiting growth/destroying cells, complementing existing therapies for numerous solid tumors.

Pioneering studies used iron–nickel alloy microdisks to trigger apoptosis in U87 cells using a low-frequency (20 Hz) 1T magnetic field *in vitro* and *in vivo*.^13^ Since then, numerous particle types, field applicators, material types, and cell types have been trialed. This includes electrodeposited iron nanowires, which showed 20–40% reductions (depending on coating) in the viability of colon cancer cells upon application of a 1 mT 10 Hz alternating magnetic field (AMF).^14^ The same study showed synergistic effects and much higher reductions in cell viability by coadministration of doxorubicin.^14^ A second example, using nickel electrodeposited nanowires, showed a reduction in cell viability of 38% (at 1 kHz) and 24% (1 Hz), where each used a 0.5 mT magnetic field.^15^ Much smaller nanostructures (*e.g*., 20 nm ZnO cubes under a rotating magnetic field at 15 Hz and 40 mT, as described in ref 16) have also been designed to target intracellular organelles including mitochondria and lysosomes. Discovery of the frequencies with the magneto-mechanical effect and its application alongside RT creates an opportunity to develop a new class of more efficacious therapy to destroy tumors using locally administered nanowires.

The aim of this work is to produce iron–gold nanowires for combined MMT and locally enhanced RT of GBM *in vitro*. The hypothesis is that magnetic iron-containing nanowires mechanically rotate, inducing membrane damage and the gold scatters X-rays due to its high atomic number, enhancing the local RT dose; thus, the combination of the two therapies increases the potency of the localized treatment (described in Figure 1). Nanoparticles are well known for their potential in multimodal cancer therapy (reviewed in ref 17), but combined MMT and locally enhanced RT of GBM using gold–iron nanowires has not previously been studied.

**Figure 1:**
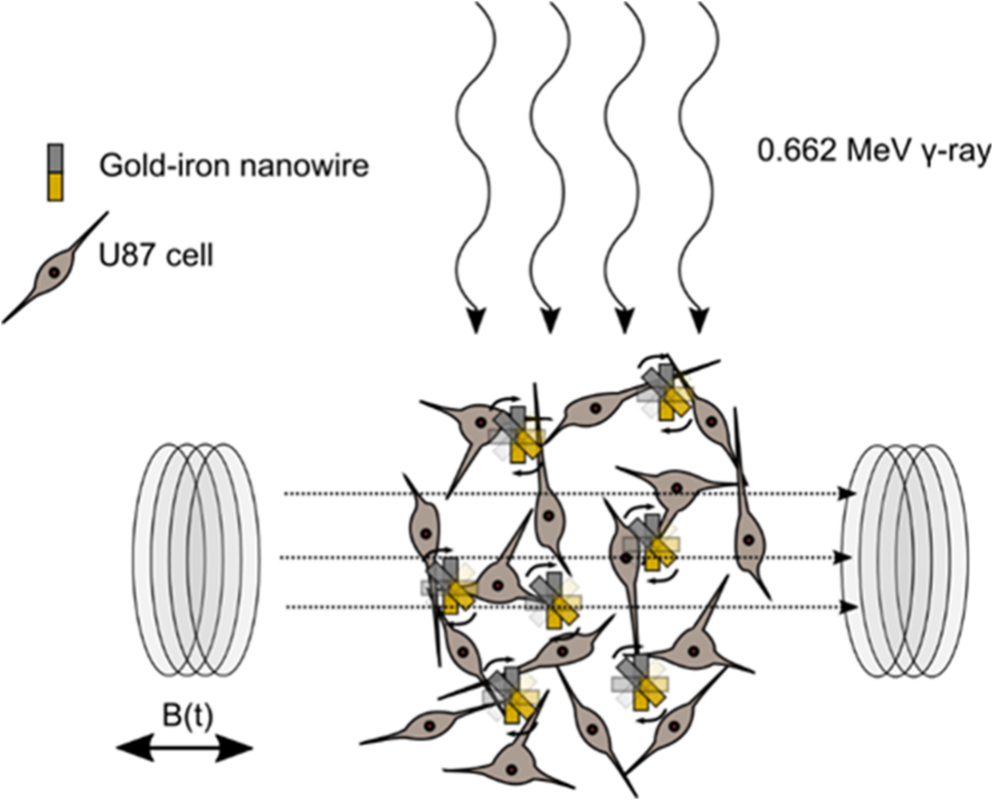
Schematic representation of U87 cells incubated with AuFe NWs and then exposed (separately) to an AMF and a 0.662 MeV Cs-137 source.

## Results and Discussion

### Development and Characterization of AuFe NWs

The first aim was to produce and precisely characterize AuFe NWs so that their dimensions and magnetic properties may be determined and used as input parameters for modeling the nanowires *in silico*.

Building on known methods (see the Experimental (Methods and Materials) section), an electrochemical method made use of a solution containing both iron and gold species as a means of selectively depositing gold and iron by varying the deposition current. A linear sweep voltogram (LSV) analysis (Figure 2A) revealed two peaks (−0.72 and −1.1 V), which indicates that a change in the applied deposition potential will change the material deposited. Correspondingly, it is expected that a change in the deposition current will change the material deposited.

**Figure 2:**
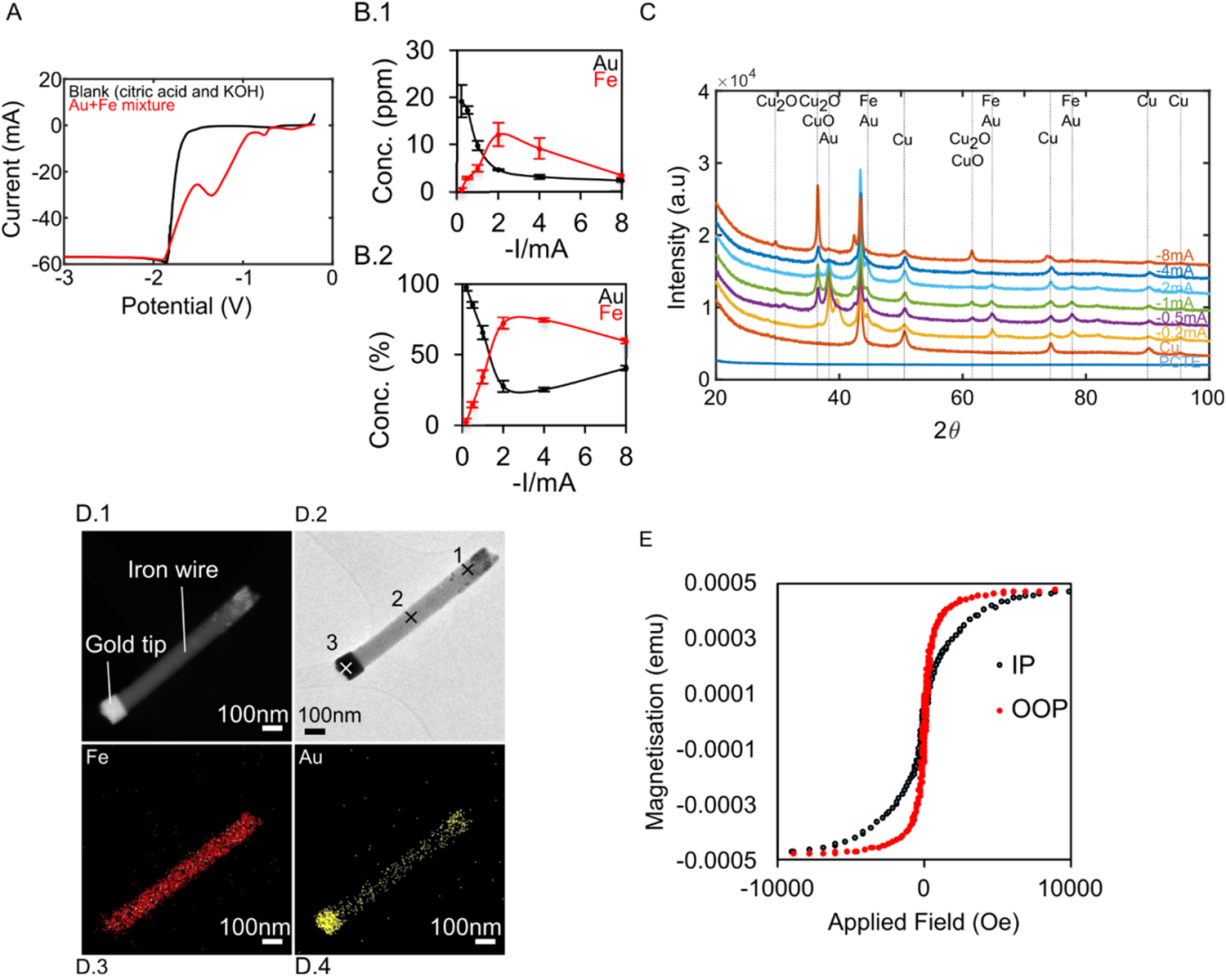
Tuning of AuFe NW electrodeposition and characterization. (A) Linear sweep voltammetry (50 mV/s) of gold–iron deposition solution. (B.1) ICP-OES quantification of gold and iron content in nanowires deposited at a range of deposition currents (1C of charge deposited at each current value) (B.2). ICP-OES quantification of (B.1) expressed as a percentage of total iron and gold in parts per million expressed in (B.1). Error bars in (B1.1, B1.2) are standard deviations (*n* = 2). (C) XRD showing patterns for a range of deposition currents. (D.1) HAADF-STEM, (D.2) Bright-field TEM, (D.3) EDX mapping (iron), and (D.4) EDX mapping (gold) showing an example AuFe NW. (E) Vibrating sample magnetometry (VSM) showing in-plane (IP) and out-of-plane (OOP) measurements of AuFe NWs.

To check this hypothesis, and to obtain detailed characteristics of the nanowires, a range of deposition currents (between −0.2 and −8 mA) were applied and analyzed using inductively coupled plasma optical emission spectroscopy (ICP-OES), X-ray diffraction (XRD), and transmission electron microscopy (TEM). A brief preliminary qualitative analysis, bringing a small Neodymium N52 magnet in close proximity to the deposits, showed the −2 and −4 mA deposits respond to the external magnet, while the −8 mA deposits respond weakly and the less negative current deposits (−0.2, −0.5, and −1 mA) did not respond to an external magnet at all.

The ICP-OES showed that as the deposition current is lowered, the proportion of iron increases, reaches a maximum, and then falls again (Figure 2B). The deposits formed at −0.2 mA had the highest gold content (96% Au, 4% Fe), while the −2 mA deposits had the highest iron content (25% Au, 75% Fe) (Figure 2B.2). Therefore, there is an optimum window at around −2 mA for iron deposition, which was adopted for preparing the AuFe NWs. As such, AuFe NWs, as they will be referred to henceforth, refer to nanowires formed by a 500s, −2 mA deposit. The fall in the proportion of iron at the lowest currents is because at these large current values, most of the deposition current, is due to hydrogen evolution at the working electrode. This results in a fall in the total amount of material deposited in absolute terms (Figure 2B.1). Various groups have reported on the effects of hydrogen side reactions leading to pH changes and morphology changes as a result of bubbles.^18,19^

The crystallographic properties of the nanowires were characterized by XRD, which revealed that the presence of: fcc metallic copper (as expected from the working electrode: peaks at 43, 50, 74, 90, and 95° (fcc)—PDF 00–004–0836), fcc gold (strongest peaks at 38, 44°—PDF 00–004–0784) (Figure 2C), bcc iron (44, 65, and 82°—ICSD 01–085–1410), and Cu_2_O (peaks at 37, 62°—ICSD 01–077–0199). The polycarbonate template alone showed no peaks between 20 and 100°, meaning that the template does not interfere with the analysis of the metal deposits.

Next, the dimensions and nanostructures of the AuFe NWs (−2 mA, 500s) were characterized using high-angle annular dark field transmission electron microscopy (HAADF-TEM) and bright-field (BF) TEM (TEM) (see Figure 2D.1,D.2, respectively) with energy-dispersive X-ray spectroscopy (EDX) (Figures 2D.3,D.4, S1 and S2). The HAADF-STEM (in *Z* contrast) revealed (referring to the particle in Figure 2D.1 and describing from the top right to the bottom left) an iron section containing gold nanostructures, a long iron section, and a gold tip. It is noted that the nanowire deposits on the copper electrode and grows along its length from region 1 to region 3. It was hypothesized that the gold-tip forms by galvanic displacement of iron atoms in the deposit by gold ions in the solution.

The gold/iron ratio of the three regions (labeled 1 to 3 in Figure 2D.2) were quantified by EDX (see Figure S1) which revealed that the gold nanostructure in region 1 contained 53/47% gold/iron, the long iron section contained 16/84% gold/iron, and the gold tip contained 80/20% gold/iron by number. For the purposes of modeling the nanowires, the particles are treated as having a length of iron and a length of gold, and consequently, the length of regions 1 and 2 together was taken as the length of iron *L*_Fe_, and the length of region 3 were taken as the length of gold (*L*_Au_). *N* = 131 individual nanowires were measured using bright-field TEM and Figure S2 shows the resulting size distributions of the total length *L* (the length of regions 1, 2, and 3), *L*_Au_ (the length of region 3), and *d* (the diameter) (note that *L*_Fe_ + *L*_Au_ = *L*), from which it was derived (peaks of the distribution were taken as averages) that *L* = 1900 nm, *L*_Fe_ = 1860 nm, *L*_Au_ = 42 nm, and *d* = 81 nm. SEM was used to visualize multiple nanowires at low magnification to confirm the homogeneity of the nanowires (Figure S3). These dimensions (*L* = 1900 nm, *L*_Fe_ = 1860 nm, and *d* = 81 nm) were used for the purpose of modeling the AuFe NWs in response to an externally applied magnetic field to determine what magnetic field parameters to use in the U87 cells.

Finally, the magnetic properties of the nanowires were quantified, to determine the magnetization parameters to input into the modeling. Figure 2E shows vibrating sample magnetometry measurements for the nanowires. Both in-plane (IP) and out-of-plane (OOP) measurements were taken for each sample. The nanowires are long cylinders with the long axis perpendicular to the plane, so OOP measurements are along the nanowire long axis.

Due to the shape anisotropy of the nanowires, the long axis of the nanowires is expected to become magnetized more easily than the direction perpendicular to the nanowire axis,^20^ which is observed (see the steeper gradient of the OOP curve compared with the IP curve in Figure 2E). The saturation magnetizations in the AuFe NWs were determined to be (145 emu/g), by normalizing to the mass of iron in the sample, measured by ICP-OES (derived from the data in Figure 2B.1), which is lower than the saturation magnetization of bulk iron (217.6emu/g). This is expected in nanomaterials because of the high surface-to-volume ratio, which results in reduced magnetic moment due to spin-disorder and oxidation at the surface of the material.^21^ The coercive field in the direction of the OOP of 92 emu is around 2 orders of magnitude above the coercivity of bulk iron (2 Oe) (derived from data near the origin in Figure 2E). The high coercivity of the nanowires arises due to the shape anisotropy of the nanowires. The saturation magnetization of 145 emy/g was used for the purpose of modeling the AuFe NWs.

### *In Silico M*odeling of Angular Trajectory of AuFe NWs in Response to Externally Applied AMF to Determine a Frequency for *In Vitro* MMT

Due to a competition between magnetic and viscous forces, the response of a magnetic nanowire to an AMF is expected to vary with the frequency of the AMF. As such, the second aim was to determine a frequency for applying MMT by *in silico* modeling, taking (1) the nanowire characteristics derived above and (2) the properties of the magnetic field applicator and viscosity discussed below as input parameters. The determined frequency was used as a test frequency for MMT *in vitro*.

The magnetic field applicator (shown schematically in Figure 3A) applies a horizontal magnetic field of the form *B*_*x*_sin­(2π*ft*) (shown schematically in Figure 3C), where *B*_*x*_ is the amplitude of the magnetic field, *f* is the frequency, and *t* is time. *B*_*x*_ was measured to be 0.76 mT, and this value of *B*_*x*_ was used as an input parameter for the modeling. The viscosity of T98G human brain cancer cells has been measured by Margraves et al. to be 76.4 times larger than water (68 mPas) for 500 nm vesicles, and this value was used in this study as an estimate of the viscosity in U87 cells.^22^ Due to the interaction between nanoparticles with the cytoskeleton, the viscosity of nanoparticles within cells depends on the cell type and on the size of the nanoparticles.^23^ We justify our assumption based on the study by Margraves et al.: (i) the 500 nm vesicles are of the same size order of magnitude as the nanowires in the present study, and (ii) T98G cells and U87 cells both are types of GBM cells.

**Figure 3:**
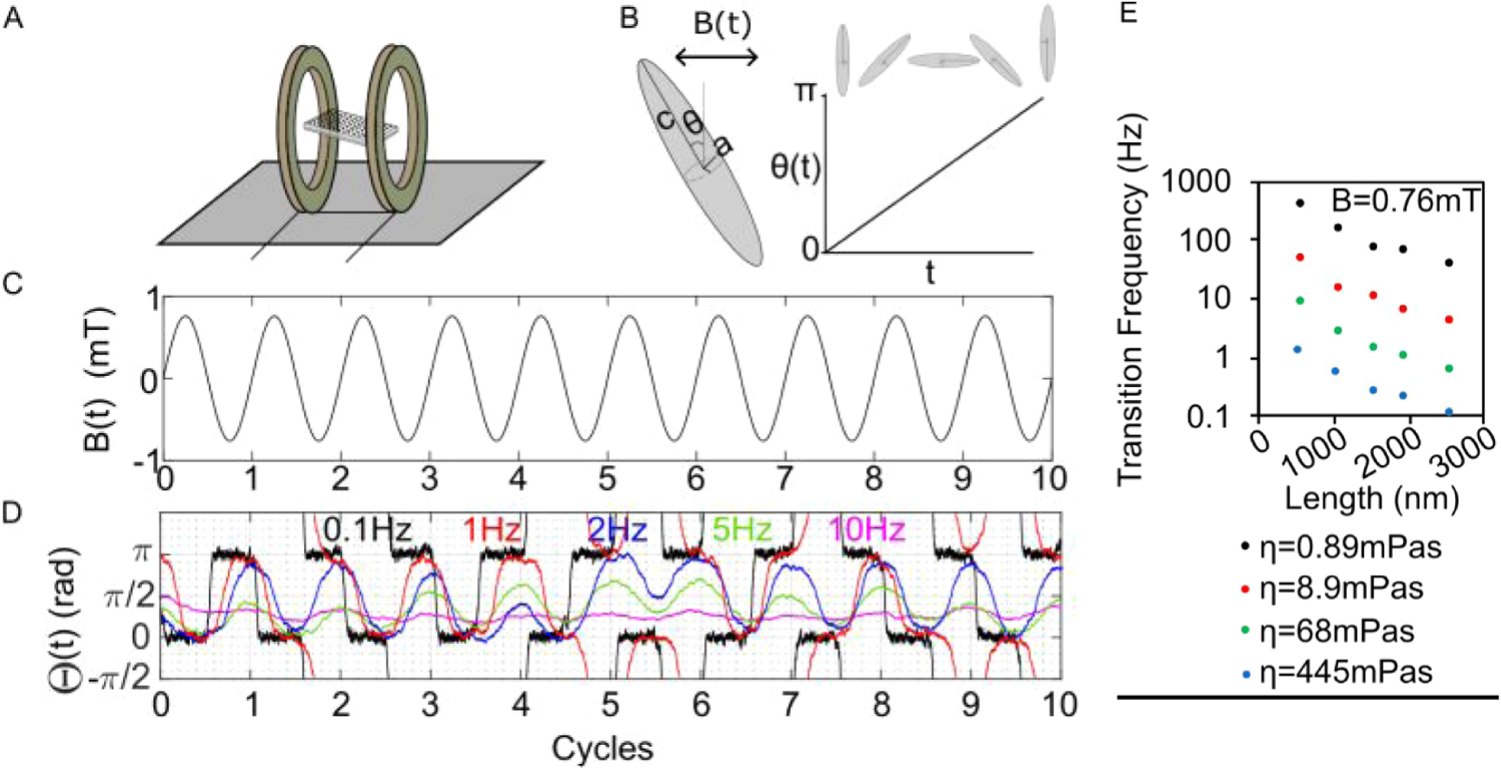
(A) Schematic representation of the AMF applicator (field applied in the horizontal direction). (B) Schematic representation of a modeled prolate ellipsoid and angular trajectory. (C) Example form of the applied AMF. (D) Example modeled nanowire trajectories at a range of applied field frequencies (0.1–10 Hz). (E) Transition frequencies of nanowires (*i.e*., the frequency at which the reorientations stop).

Figure 3B shows a schematic diagram of the modeled nanowire trajectory: the nanowire in Figure 3B is moving from 0 to π radians, undergoing a reorientation. Figure 3D shows the modeled angular trajectories for *B*_*x*_ = 0.76 mT for a nanowire with (*d* = 80 nm, *L* = 1900 nm, *M*_s_ = 145 emu/g) at a range of frequencies between 0.1 and 10 Hz. At 0.1 Hz, the nanowires move with a square waveform at a frequency of 0.1 Hz, indicating that the particles are undergoing complete reorientations in time with the time-varying magnetic field, and then remain in their reorientated position until the external magnetic field reverses in direction. At 1 Hz, the square waveform is no longer observed as the increased frequency means the particle only just completes a reorientation in the time available before the magnetic external magnetic field reverses in direction. Between 2 and 10 Hz, the nanowire increasingly fails to reorientate fully (*i.e*., does not reach 0 or π radians when reorientations occur), and at 10 Hz, very little angular movement is observed.

For MMT, we hypothesized that it is desirable for the nanowires to be in motion as much as possible, which means that neither the low-frequency behavior (*e.g*., as in Figure 3D at 0.1 Hz, where the nanowires remain static in their reorientated position between reorientations) nor the high-frequency behavior (*e.g*., as in Figure 3D at 10 Hz where the nanowires remain static and fail to reorientate) is desirable. At intermediate frequencies (*e.g*., as in Figure 3D at around 1 Hz), the largest amount of continuous motion is observed because the nanowires consistently reorientate, but do not stay static between reorientations.

To identify a target frequency for MMT, the frequency at which a modeled nanowire fails to reorientate to within π/10 of 0 or π radians more than 1 in 10 times was recorded. This frequency was recorded for a range of nanowire lengths and is referred to as the “transition frequency” and denoted *f*_T_. In the example shown in Figure 3D, for a nanowire with (*d* = 80 nm, *L* = 1900 nm, *M*_s_ = 145 emu/g), *f*_T_ was determined to be 1.1 Hz (the decimal point was determined by testing granularly between 1 and 2 Hz, but is not shown for simplicity of the diagram). Above *f*_T_ (*e.g*., as shown in Figure 2D at cycle 4 for the blue 2 Hz trajectory), reorientation failure starts to be observed.

Figure 3E shows a plot of transition frequencies for a range of nanowire lengths and at a range of fluid viscosities, where each data point was identified by a process similar to that explained for Figure 3D. *f*_T_ decreases with nanowire length and fluid viscosity. For example, *f*_T_ for 2000 nm nanowires at η = 68 mPas was determined to be 1 Hz. Because only very few (1.5% of nanowires, or 2 nanowires in *N* = 131 sample) had lengths greater than 2000 nm, a frequency of 1 Hz was adopted for *in vitro* testing. As such, the majority of the nanowires (98.5% of which are shorter than 2000 nm) can be expected to consistently reorientate at 1 Hz.

### *In Vitro* Study of the Effect of AuFe NWs on MMT and RT (Separately and in Combination) in U87 Cells

The third aim was to determine whether AuFe NWs can increase the efficacy of RT or MMT. As such, the AuFe NWs were tested in combination with RT, MMT, and a combination of RT and MMT.

Before using the nanowires for radiotherapy or MMT, the maximum dose of nanowires that did not cause an adverse response was determined. AuFe NWs were administered to cells at a range of concentrations, showing that higher concentrations AuFe NWs (above 0.2 μg/well) lead to viability below 80%, as determined by MTS assays (Figure S5). The AuFe NWs caused a reduction in cell viability as high as the positive control (to 34 ± 7%) at 2 μg/well. To minimize toxicity effects from the AuFe NWs alone, doses of 96 ng per well (administered using 100 μL nanowires at 0.96 μg/mL) were used for subsequent experiments as a threshold dose that does not cause a significant reduction in viability of these cells. This corresponds to around 200 nanowires per seeded cell.

Next, U87 cells incubated with and without AuFe NWs were irradiated with a range of doses (0–20 Gy) of 0662 MeV Cs-137 RT (Figure 4A). At 20 Gy, the viability measured by MTS assay 72 h post seeding was (69 ± 6)% (without AuFe NWs), which corresponds with a ∼30% drop in cell viability in the irradiated cells, which was significantly different to the control nonirradiated cells (*p* = 0.002, *t* test) (Figure 4A). This ∼30% reduction in cell viability may be attributed to the 20 Gy 0.662 MeV RT. There was no significant difference (*p* = 0.219, *t* test) observed between the cells with/without AuFe NWs. This suggests that AuFe NWs do not increase the efficacy of doses between 0 and 20 Gy of 0.662 MeV RT on U87 cells.

**Figure 4:**
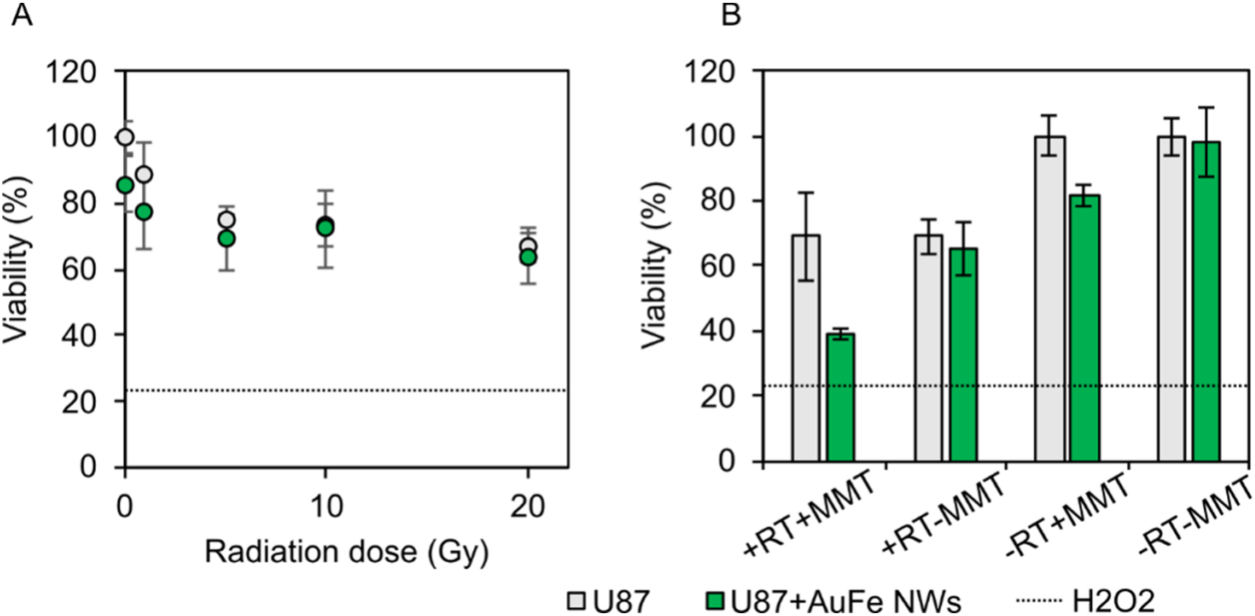
Cell viability of U87 cells following various therapies measured by MTS assay 72 h post cell seeding (*n* = 4), error bars are standard deviations. (A) U87 cell viability following exposure to 0.662 MeV Cs-137 RT at various doses at ∼4 h post seeding. (B) U87 cell viability following exposure to 0.662 MeV Cs-137 RT (20 Gy) (denoted + RT) and/or MMT for 1 h at 1 Hz using a 1 Hz 0.76 mT AMF (denoted + MMT). Dotted lines indicated cell viability in response to the 1% H_2_O_2_ positive control.

Second, U87 cells incubated with AuFe NWs were exposed for 1 h to a 1 Hz 0.76 mT AMF (Figure 4B, compare −RT-MMT with −RT + MMT). As explained in the previous section, this frequency and field strength were shown using the modeling to induce rotations in the AuFe NWs at viscosities which are expected in the cells. In the cells with AuFe NWs, following application of the AMF, a cell viability measured by the MTS assay 72 h post seeding of (82 ± 3)% was observed, corresponding with an ∼20% reduction in cell viability (*p* = 0.007, *t* test) (compare the green bar above −RT + MMT with the green bar above −RT-MMT).

There was no evidence of a reduction in cell viability from the AuFe NWs alone (cell viability of (98 ± 10)% at 72 h) or from the application of the magnetic field alone (cell viability of (100 ± 6)% at 72 h). Therefore, the aforementioned evidence provides that the magnetic field facilitates an interaction between the nanowires and the cells, which drives a reduction in the cell viability of ∼20%.

Third, we investigated the cell viability after applying a 1 Hz AMF followed by 20 Gy RT for cells incubated with or without AuFe NWs (Figure 4B). In these conditions, the cell viability of U87 cells incubated with AuFe NWs at 72 h was (39 ± 2)%, corresponding with a ∼60% drop in cell viability (see the green bar above + RT + MMT). By contrast, the control U87 cells (without AuFe NWs, but still with the magnetic field and radiation applied) had a cell viability at 72 h of (69 ± 14)%, which is accounted for by the 30% drop expected following the 20 Gy 0.662 MeV RT (see the gray bar above + RT + MMT). To investigate if the difference in the effect (*i*.*e*., 60% when combining RT and MMT versus 30% from RT alone, or 20% from MMT alone) could be attributed to an interaction of the RT and MMT, a 2 × 2 ANOVA using Welch statistics was performed. This analysis showed evidence of an interaction effect between the RT and the MMT when the cells had been incubated with AuFe NWs (*p* = 0.01, ANOVA). No interaction was observed without the AuFe NWs (see the gray bar above + RT + MMT). This strongly suggests that the MMT drives an association between the nanowires and the cells (which in turn influences the radiosensitization).

Summarizing, AuFe NWs (without MMT) do not increase the efficacy of a 20 Gy 0.662 MeV RT. However, AuFe NWs (following application of a 1 Hz 0.76 mT AMF for 1 h) do increase the efficacy of 20 Gy 0.662 MeV RT from around a 30% reduction (without AuFe NWs) to around 60% (with AuFe NWs). Therefore, there is a response of the AuFe NWs to a 1 Hz 0.76 mT AMF, which causes: (i) a reduction in cell viability and (ii) an increased efficacy of RT.

### Quantification of Cellular Association of AuFe NWs with U87 Cells

We investigated our hypothesis that the MMT drives an association between the nanowires and the cells by studying the positions of the nanowires within the cells using Prussian blue to stain the iron in the nanowires and visualizing their locations using an optical microscope (Figure 5A). Blue regions (Prussian blue-stained iron) against a pink background (Nuclear Fast Red counter stain) correspond with regions where AuFe NWs have become associated with the U87 cells. We made the following visual observations: (i) there are no iron deposits (blue) in the cells which had not been incubated with AuFe NWs (see Figure 5A, top row), which means that there are no other perceptible contributions to the iron regions other than from the AuFe NWs, (ii) there was a marked increase in the proportion of the cells stained blue following application of the magnetic field (compare + MMT and −AMF on the bottom row at 24 and 72 h), which suggests cell membrane association/internalization of the AuFe NWs with the cells is more effective once the MMT has been applied, (iii) blue deposits (iron) are observed following/despite washing and fixing steps, which indicates that the iron is either associated with the plasma membrane of the cells or has become internalized, and (iv) the proliferation of cells incubated with gold iron nanowires is affected by the application of the magnetic field (compare + MMT (which has proliferated less, and has more iron deposits) with −MMT (which has proliferated less, and has fewer iron deposits) at 72 h). The final observation suggests that the association of the AuFe NWs with the cells, and their subsequent actuation by the magnetic field may play a role in preventing the proliferation of the U87 cells. The proliferation was quantified by the MTS assay discussed in Figure 4B, which supports the same conclusion.

**Figure 5:**
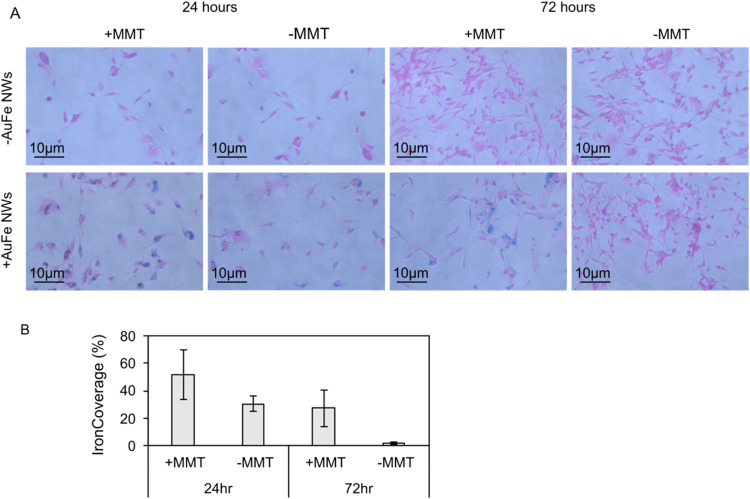
Imaging and quantification of AuFe NW association with the cells. (A) Conventional optical microscopy showing cells (pink) and iron (blue) for U87 cells incubated with and without AuFe NWs and subjected to an AMF field at 24 and 72 h time points. (B) Quantification of percentage of iron associated with the cells (*i.e*., blue/(blue + pink)) for the range of conditions shown in (A).

The % iron coverage was quantified (Figure 5B). This parameter represents the quantity of iron associated with the cells. At the 24 h time point, the % iron coverage was (52 ± 18)% following application of a 1 Hz 0.76 mT AC magnetic field for 1 h at the 1 h time point, compared with (31 ± 5)% in the cells to which no magnetic field was applied. This represented a significant increase (*p* = 0.044, *t* test) in association of iron with the cells, which corresponds with an increase in association of AuFe NWs with the cells, driven by the application of the magnetic field. At the 72 h time point, the % iron coverage was (27 ± 14)% following application of a 1 Hz 0.76 mT AC magnetic field for 1 h at the 1, 24, and 48 h time points, compared with just (2 ± 1)% in the cells to which no magnetic field was applied. This increase in association of AuFe NWs with the cells was also significant (*p* = 0.017, *t* test). The lower association of iron with the cells at 72 h as compared with at 24 h is due to the proliferation of cells by 72 h, which results in a greater number of cells in which there is no association of AuFe NWs. As noted above, the cell proliferation is inhibited by the AuFe NWs actuated by the magnetic field, which explains why there is a larger effect size seen at 72 h than at 24 h. This cell-nanowires association could facilitate radiosensitization, enabling reactive oxygen species (ROS) generation or local scattering processes by incident radiation, around or inside the cells, to enhance their damaging effects.

To understand the interaction of the nanowires with the cells, scattering-type scanning near-field optical microscopy (s-SNOM) and atomic force microscopy (AFM) were used to produce high-resolution images of nanowires within cells. s-SNOM is a relatively new technique for imaging of nanoparticle interactions with cells.^23^ S-SNOM has significant advantages over conventional high-resolution bioimaging techniques like TEM due to the much more difficult sample preparation (including the challenge of ultramicrotoming thin sections of cells containing hard Fe–Au nanowires).

An optical microscope operating in reflection mode was used to search for the nanowires in the cells before imaging them with s-SNOM and AFM (Figure 6A). AuFe NWs can be identified by their topography (AFM) or by their s-SNOM amplitude, which is large in comparison to that of organic material. Without the application of a magnetic field (−AMF) (top row of Figure 6A), there is minimal disturbance to the cell membrane at the locations of the NWs. With the application of a magnetic field (+AMF) (bottom row of Figure 6A), however, there is significant disturbance of the cell membrane, with a large amount of material piled on top of the NWs. This material appears to be organic from its s-SNOM amplitude, and is probably debris from the cell membrane. It is thus concluded that the NWs have caused significant damage to the cell membrane under the applied magnetic field.

**Figure 6:**
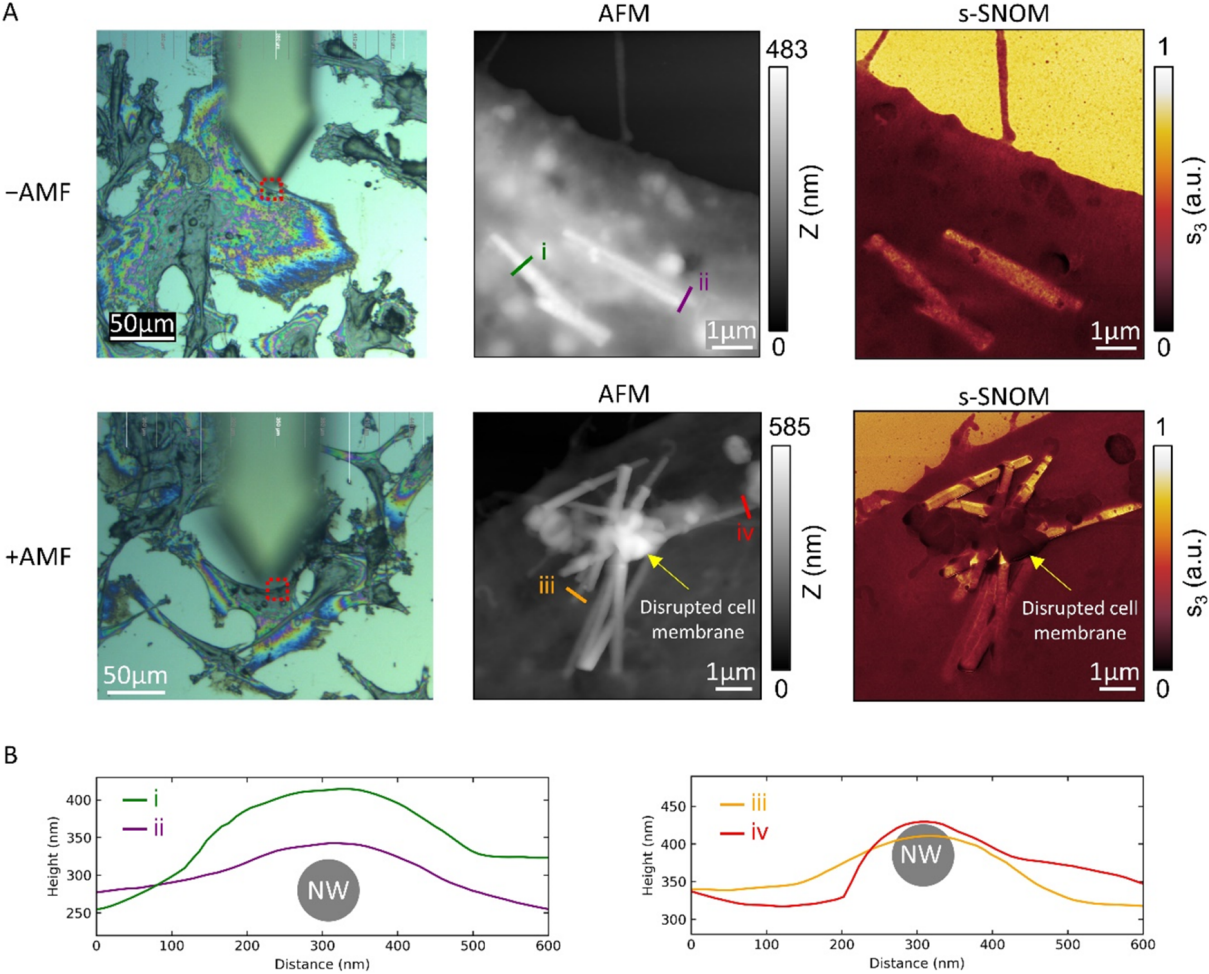
(A) Left: Conventional (optical) microscopy image of U87 cells on an Si chip with (bottom) and without (top) a magnetic field (AMF), taken using a built-in optical microscope on an s-SNOM system. Red boxes show s-SNOM and AFM scanning areas. Centre and right: AFM (topography) and s-SNOM (third harmonic optical amplitude) images show nanowires interacting with cells. The illumination wavenumber was 1650 cm^–1^ to target amide functional groups. In the + AMF case, there is disruption to the cell membrane observed in the AFM and s-SNOM images. (B) Topography profiles along the lines shown in (A) with 81 nm diameter NW cross section shown as a guide. The curvature of the profiles suggests that NWs i-iii are beneath the cell membrane, while iv is above it. This is congruent with the larger s-SNOM amplitude measured for NW iv.

Another advantage of using s-SNOM/AFM is that topographical profiles extracted from AFM images provide a means of confirming that the AuFe NWs are internalized by cells. For example, the profiles i–iii shown in Figure 6B have a curvature much larger than the diameter of NWs (a cross section of an 81 nm diameter NW is shown for reference). The NWs in these locations must, therefore, be below the cell membrane. The profile iv, on the other hand, has a curvature that is more consistent with the NW diameter (allowing for the apparent broadening of features comparable in size to the finitely sharp tip of the imaging probe). The NW in this location is likely to be above the cell membrane. This is supported by the large s-SNOM amplitude in this location, which is expected for a bare metallic surface but not for organic material.

In this study, a frequency for performing MMT was selected by *in silico* modeling based on measured dimensions and magnetic properties of gold nanowires. The frequency selected by modeling was evaluated *in vitro*. As such, this study represents a proof-of-concept of a principled approach to designing treatment regimens for MMT. In summary, AuFe NWs were measured to have (i) average dimensions of *L* = 1900 nm, *L*_Fe_ = 1860 nm, and *d* = 81 nm, (ii) a saturation magnetization of 145 emy/g. Based on these measured parameters, *f*_T_ was determined to be 1.1 Hz. As explained above, *f*_T_ represents a frequency above which the modeling predicted that reorientations of the AuFe NW happen with an increasingly reduced probability and below which the AuFe NW remains increasingly static between reorientations. Therefore, MMT at *f*_T_ increases the chance of the AuFe NW being in rotational motion compared to higher or lower frequencies. Finally, rotations of the AuFe NW were induced *in vitro* in U87 cells (and in combination with RT) which resulted in a 20% reduction in U87 cell viability (MMT alone) and a 60% reduction in cell viability (*p* = 0.01, ANOVA) (in combination with 0.662 MeV Cs-137 RT).

Three mechanisms could cause the observed reduction in cell viability associated with MMT and RT. First, an association of the nanowires with the cells (which increases following application of the magnetic field; see Figures 5 and 6) may create cell-nanowire contact, which drives a synergy between the MMT and RT effects observed in the cells. The synergy may be explained because the distance between the nanowires and the cells and the relative position of the nanowires within the cells affect the damage caused by the secondary scattering of the radiation by the nanowires.^9^ Therefore, we speculate that the MMT drives an association of the nanowires with the cells, which increases damage caused by secondary scattering of the radiation by the nanowires due to increased proximity between the nanowires and the cells. Second, rotation of the nanowires under MMT could also induce membrane damage and/or lysosome permeabilization.^24^ These processes lead to disorganization of the tubulin microtubule network during MMT and a change in cell morphology, cell adhesion, and ultimately cell apoptosis.^25^ Third, internalization of the nanowires could also cause increased ROS within the cells, which leads to oxidative stress.^10,26,27^

Compared with previous studies, *e.g*., Martinez-Banderas et al.,^14^ which showed synergistic effects of MMT of electrodeposited iron nanowires in combination with doxorubicin at a field strength 1 mT and frequency of 10 Hz, MMT was here used for the first time in combination with RT, which increased the reductions in cell viability from 20% (MMT alone) to 60% (MMT in combination with RT). Thus, this study provides evidence that MMT can also complement existing therapies of GBM, such as RT. A study by Contreras^15^ showed a reduction in cell viability of 24% at 1 Hz for 0.5 mT MMT of electrodeposited Nickel nanowires in HCT116 cells (having 30–40 nm diameters and ∼4 μm length). Similarly, our results showed a 20% reduction in cell viability at 1.1 Hz, which our modeling suggested is indicative of a regime where the nanowires have an increased chance of being in rotational motion. We then increased the therapeutic efficacy by combining MMT with RT.

Limitations of the present study include: (i) the modeling does not have a direct measurement of the viscosity of U87 cells (and we therefore assumed that U87 cells behave analogously to T98G cells as measured to be 68 mPas by Margraves et al.^22^), (ii) we did not observe the motion of the AuFe NWs directly. Future work could provide further validation of the modeling by carrying out direct observations (*e.g*., by fluorescence labeling AuFe NWs and live-cell imaging). In addition, future *in vivo* studies should aim to conjugate therapeutic targets overexpressed on the cell surface of GBM cells, such as to the EGFR or EphA3 receptors,^28,29^ to mitigate any possible unwanted off-target toxicity and to improve nanowire targeting to GMB cells *in vivo*. The *in vivo* biodistribution of the nanowires should also be assessed. A further limitation is that not all of the conditions simulated *in silico* were validated *in vitro*. The validity of *in silico* models should be interrogated further *in vitro* (*e.g*., testing a wider range of magnetic field strengths and frequencies) to confirm their ability to make realistic *in vitro* predictions.

## Conclusions

Regarding our aims and hypotheses as expressed in the introduction, we have produced iron–gold nanowires for combined MMT and locally enhanced RT of GBM *in vitro*. Magnetic iron-containing nanowires have been shown by *in vitro* modeling to mechanically rotate, and *in vitro*, reduce cell viability, which we attributed to associations between the AuFe NWs and U87 cells. Using AFM and s-SNOM, we observe that the AuFe NWs induce local membrane damage and the gold scatters X-rays due to its high atomic number, enhancing the local RT dose. The combination of the two therapies increases the potency of the localized treatment from a 20% reduction (MMT alone) to a 60% reduction (MMT and RT). This represents the first trial of a combination therapy of MMT and RT. The results are a basis for a new class of treatments utilizing the combination, which would be relevant to various solid tumors since the damage induced by MMT and RT is not expected to be specific to the U87 cells studied here. Developing MMT founded on a rational calculation of the required frequency based on measurements of particle dimensions and magnetic properties is an approach that could accelerate MMT development since conditions can be screened more quickly *in silico* prior to *in vitro* testing. The AuFe NWs have other orthogonal therapeutic and diagnostic potential uses such as in magnetic resonance imaging (MRI), magnetic hyperthermia, contrast enhancing (making use of the magnetic properties of the iron), and surface-enhanced Raman spectroscopy (SERS) imaging and photothermal therapy (making use of the plasmonic properties of the gold), which could be utilized in the future to improve patient outcomes clinically.

## Experimental (Methods and Materials)

### Synthesis and Characterization of AuFe NWs

Here, AuFe NW synthesis was achieved using an electrochemical hard-template technique by adapting earlier work by various groups (*e.g*., refs 30,31 ).

AuFe NWs were electrodeposited in the pores of a polycarbonate track-etched (PCTE) membrane (which was used as a template for nanowire synthesis and is referred to as a PCTE template). The PCTE template was coated on one side with around 100 nm copper using a Korvus Technology Hex Series Modular Deposition System to serve as a working electrode. For carrying out the nanowire depositions within the PCTE template, it was then assembled in a three-electrode system with the electrodes connected to an Ivium Technologies Compact Stat potentiostat. The same potentiostat and electrodes were used to carry out linear sweep voltammetry at 50 mV/s. A Pt mesh counter electrode and a 3 M Ag/AgCl reference electrode were used. The PCTE template was incubated for around 1 h with DI water to facilitate wetting of the pores. The DI water was removed, replaced with 1 M CuSO_4_, and a 250 s–2 mA pulse was applied to deposit a sacrificial copper base layer for contacting the working electrode with the nanowires. The CuSO_4_ was replaced with a freshly prepared gold–iron solution containing 0.29 M FeSO_4_, 2.5 mM Techni gold ES, 0.49 M citric acid, and 1.76 M KOH (pH ∼ 6.15). A −2 mA 500s pulse was applied to deposit AuFe NWs. The PCTE template was immediately rinsed in DI H_2_O and dried. Techni Gold 25 ES is a neutral noncyanide gold plating solution obtained from Techni, Inc., which mixture of sulfuric acid, ethylenediamine, sodium sulfite Na_2_SO_3_, and sodium gold sulfite Na_2_Au­(SO_3_)_2_ at proprietary ratios. 2.5 mM refers to the final concentration of gold ions in the gold–iron solution. To remove the sacrificial copper and working electrode, a solution of 4 M NH_3_, 1 M NH_3_Cl, and 0.5 M CuCl_2_·5H_2_O was pipetted on the copper side, followed by thorough rinsing in DI H_2_O and drying (see Figure S4). The etching solution was prepared according to an article on selective recovery of copper from mixed copper and iron waste.^32^ The efficiency with which the etching solution removed the copper without affecting the iron was important for producing maximal yields of AuFe NWs. The dry template moved in response to being brought in the proximity of an N52 Neodymium magnet.

The gold and iron content of the deposits was analyzed by inductively coupled-optical emission spectroscopy (ICP-OES) using a ThermoScientific iCAP 6000 Series ICP Spectrometer, following dissolution of the samples in 1 mL freshly prepared concentrated Aqua Regia for at least 1 h. Samples were diluted to a final volume of 5 mL in ultrapure water for analysis. Percentages of gold and iron (see Figure 2B.2) were obtained according to 100 × *C*_Au_/(*C*_Au_ + *C*_Fe_) and 100 × *C*_Fe_/(*C*_Au_ + *C*_Fe_) for gold and iron, respectively, where *C*_Au_ and *C*_Fe_ are the concentrations of gold and iron in ppm obtained from the ICP-OES analysis, respectively. Transmission electron microscopy (TEM) samples were prepared by dissolving the dried PCTE template using dichloromethane (DCM), before pipetting a small (∼5 μL) droplet of the suspended nanowires onto 300 mesh holey carbon copper grids (TAAB). Scanning TEM, TEM, and energy-dispersive X-ray spectroscopy (EDX) compositional analysis were performed using a JEOL JEM-2100F TEM. For SEM, the Au samples were prepared by pipetting a small (∼5 μL) droplet of the suspended nanowires onto a silicon wafer attached to an aluminum stub by carbon tape and analyzed using a LEO Gemini 1525 field emission gun scanning electron microscope. XRD was carried out using a Panalytical X’Pert multipurpose diffractometer. PCTE templates containing nanowire deposits were taped to a silicon zero-background sample holder. Vibrating sample magnetometry (VSM) was performed by using a Quantum Design vibrating sample magnetometer for a physical property measurement system (PPMS). In-plane (IP) and out-of-plane (OOP) magnetic field loops (M-H loops) were measured at room temperature (300 K) with magnetic fields of up to 3 T applied. Samples were cut down to approximately 3 mm × 3 mm, and the same cut was used for both IP and OOP measurements. The OOP measurements used a copper sample holder that demonstrates a diamagnetic response, and the M-H loops were corrected for diamagnetism.

### Preparation of Nanowires for Administration to Cell Cultures

To prepare the nanowires for *in vitro* administration, 10 μL each 25 mM methoxy PEG thiol (M.W. 5000 Da, NANOCS, Inc.) and 25 mM methoxy poly­(ethylene glycol) (PEG) phosphate (M.W. 5000 Da, JenKem Technology) in dimethyl sulfoxide (DMSO) was added to a 1.5 mL Eppendorf tube containing 0.5 mL of ethanol and a 3 mm × 3 mm piece of the nanowire template. 1 mL of DCM was then gradually added (20 μL every 30 s) and gently vortexed until the template broke up, dispersing the nanowires. Adding DCM too quickly caused irreversible agglomeration of the nanowires. The nanowires were collected using a strong Neodymium (N52) magnet, and the nanowires were rinsed in DCM and ethanol, and finally suspended in Dulbecco’s phosphate-buffered saline (pH 7.0–7.3) (no calcium, no magnesium) (DPBS) (supplied by Gibco) under a sterile laminar flow biosafety cabinet. The nanowires were again collected using a magnet, DPBS was removed, leaving a 50 μL droplet, which was sonicated to disperse the nanowires. 1 mL of complete culture medium (Dulbecco’s modified Eagle’s medium (DMEM) + 10% fetal bovine serum (FBS) + 1% pen/strep) was added, and the nanowire suspension was then diluted 1:10 in complete culture medium.

### Modeling of Nanowire Response to Magnetic Field

MATLAB calculations were performed by using MATLAB R2018b. The saturation magnetization of iron was taken as 1710 emu/cm^3^. The temperature was taken as *T* = 298 K in all cases unless otherwise stated. The evolution of AuFe NWs in response to an applied AMF was carried out using an equation adapted from Shine and Armstrong 1987 (see eq 1) which provides solutions for an axisymmetric ellipsoid in an applied magnetic field0.^33^ The equations developed by Shine and Armstrong were adapted to include the gold end of the nanowires using (L_Fe/L_Tot) which compensates for the difference in magnetic volume and actual volume (a volume ratio would otherwise cancel out). A magnetic field of the form *B* = *B*_*x*_ sin­(2π*ft*) was used, and the angular trajectory was calculated using

1 $$\Delta \theta =-\left(\frac{MB_{x}\sin2\pi \mathrm{ft}}{\eta }\right)\left(\frac{L_{\mathrm{Fe}}}{L_{\mathrm{Tot}}}\right)\frac{[2{\left(\frac{c}{a}\right)}^{2}\left(1-A\right)+A]\sin\theta }{4\left[{\left(\frac{c}{a}\right)}^{2}+1\right]}\Delta t\pm \gamma$$

Δθ is a step in the angular trajectory, *M* is the saturation magnetization in emu/cm^3^, *B*_*x*_ is the peak magnetic field in mT, *f* is the frequency of the magnetic field, *t* is the time (starting at 0, and extending to 10 cycles at which point the modeling terminates), η is the fluid viscosity in mPas, L_Fe is the length of the iron section of the nanowire, L_Tot is the total length of the nanowire, c/a is the aspect ratio (note 2*c* = L_Tot and 2*a* = diameter), *A* is a geometric parameter defined below, Δ*t* is the time step, and γ is the magnitude of a step in the nanorod’s rotational random walk.

A schematic diagram of the nanowire modeled as a prolate spheroid is shown in Figure 3B: the angle θ is the orientation of the long nanowire axis with respect to the externally applied magnetic field, the nanowire axes have a length 2*c* (long axis) and diameter 2*a* (short axis). The prolate spheroid shape was used to simplify modeling of the angular trajectory, as eq 1 (and also eqs 2–4) assumes the shape to be a prolate spheroid.

Since the nanorod is undergoing rotational diffusion at the same time as the magnetic field, the γ term is important. Without the rotational diffusion, the nanorod would become stuck in unstable equilibria. The initial orientation was randomly generated and then based on a series of computations using^1^ up to a termination at 10 cycles, an angular trajectory was calculated. This was repeated at a range of values of f until a “*transition frequency”* (denoted *f*_T_) was found. *f*_T_ was defined as the frequency above which the nanowire fails to reorientate at least once in each run of 5 consecutive runs, where failure to reorientate is defined as not moving to within π /10 (18°) of the fully aligned position and each run has 10 cycles. Thus, *f* = *f*_T_ represents the frequency at which the nanowires begin to become stuck, but are in fact still reorientating 90% of the time.

The geometric parameter *A* can be determined by^33^

2 $$A=\frac{{\left(\frac{c}{a}\right)}^{2}}{{\left(\frac{c}{a}\right)}^{2}-1}-\frac{{\left(\frac{c}{a}\right)}^{2}{\cos h}^{-1}\left(\frac{c}{a}\right)}{{\left[{\left(\frac{c}{a}\right)}^{2}-1\right]}^{3/2}}$$

How large the random walk steps γ should be in relation to the size of the timesteps Δ*t* can be determined as follows. By taking the size of the step in the random walk as

$$\gamma^{2}=\left\langle \theta^{2}\right\rangle =2D_{\mathrm{ax}}\Delta t$$

where *D*_ax_ is the rotational diffusion coefficient for rotations about an axis perpendicular to the long axis of the particle, and combining ⟨θ^2^⟩ = 2*D*_Ax_Δ*t* and *D*_Ax_ = 1/(2*t*_ax_) to give γ^2^ = Δ*t*/*t*_ax_ the size of the random walk steps and sampling times can be set appropriately in relation to each other.

The Perrin friction factors provide a solution for prolate spheroids and the time constant for rotational relaxation about an axis perpendicular to the long direction of the nanowire can be calculated as^34^

3 $$t_{\mathrm{ax}}=\frac{1}{2k_{B}T}\frac{16\pi \eta a\left(a^{2}+c^{2}\right)}{3}\frac{\left(p^{2}-1\right)}{(2p^{2}-1)S-p^{2}}$$

where

4 $$S=\frac{p}{\sqrt{p^{2}-1}}\;\ln\left(p^{2}+\sqrt{p^{2}-1}\right)$$

p=ca

for prolate spheroids with *c* > *a*.^31^ The symbols not introduced in previous equations are as follows: *k*_B_ is the Boltzmann constant, *T* is the temperature in *K*, and *p* is the aspect ratio. In practice, γ was set arbitrarily between 0.01 and 0.1 radians and the appropriate sampling time was then computed using γ^2^ = Δ*t*/*t*_ax_.

### *In Vitro* Cell Culturing

U87 MG human cells (denoted U87 elsewhere in this article) certified by the European Collection of Authenticated Cell Cultures were supplied by Sigma-Aldrich. The cells were shipped on dry ice and stored at −140 °C in a nitrogen vapor freezer upon receipt. The frozen stocks were rapidly thawed using a temperature-controlled water bath set to 37 °C, and the cells were added to prewarmed complete culture medium DMEM supplemented with 10% FBS and 1% penicillin/streptavidin in a 15 mL Falcon tube. DMEM (+ 1 g/L d-glucose, l-glutamine, and pyruvate) (DMEM), FBS, and penicillin/streptomycin were supplied by Gibco. The cells were pelleted by centrifugation at 130*g* for 7 min, excess medium was removed to ensure removal of DMSO in the cryopreservation medium, and the cells were resuspended in a final volume of 10 mL complete culture medium. The cells were then plated in tissue-culture treated filter cap T75 flasks (Nunclon Delta). The cells were subcultured in a 1:4–1:5 ratio every 3–5 days, washing with DPBS, and then using 0.25% trypsin-ethylenediaminetetraacetic acid (EDTA) to detach the cells from the plate. Dulbecco’s phosphate-buffered saline (pH 7.0–7.3) (no calcium, no magnesium) was supplied by Gibco, and the trypsin EDTA was supplied by Sigma-Aldrich. An aliquot of the low passage cells was retained and cryopreserved at 1–3 × 10^6^ cells/mL in DMEM supplemented to final concentrations of 10% FBS and 5% DMSO by freezing slowly to −80 °C in a Nalgene *M*_r_ Frosty freezing container before transferring the cells to −140 °C for long-term storage. DMSO was supplied by Sigma-Aldrich. Before freezing, healthy cells were quantified using 0.4% Trypan blue staining using a hemocytometer. The cells were discarded after 20 passages and replaced with cells from cryopreserved stocks. The received cells were certified free of mycoplasma, and the cells were routinely checked for fungal and bacterial contamination.

Cell viability assays were performed using (3-(4,5-dimethylthiazol-2-yl)-5-(3-carboxymethoxyphenyl)-2-(4-sulfophenyl)-2H-tetrazolium) (MTS) assay kits (Abcam). The cells were plated in tissue-culture-treated 96-well plates, and the seeding density was determined using a 0.4% Trypan blue aqueous solution (Gibco) and a hemocytometer. Cells were plated by adding 100 μL of cell suspension (in complete culture medium) to the wells, before adding 100 μL of complete culture medium. To include nanowires or H_2_O_2_, the substance was diluted into the 100 μL complete culture medium to add to the cells. The cells were subjected to a range of conditions (*e.g*., magnetic field or room temperature) and incubated for 24, 48, or 72 h after seeding in a humidified cell culture incubator at 37 °C with 5% CO_2_. Optimal seeding levels of 10,000, 7500, and 6000 were determined for 24, 48, and 72 h assays, respectively, maintaining a balance between a large signal and normal cell growth. Three to four replicates were used per condition, and error bars are the standard deviation across the replicates. 20 μL of MTS reagent at room temperature was added directly to each well, and the plates were incubated for precisely 1 h. Positive controls were prepared identically but used a final volume of 0.1% (w/v) H_2_O_2_ in each well. The plates were briefly shaken, and the absorbance was read at 490 nm by a Molecular Devices Spectra Max plate reader.

A background (BG) measurement for each plate was made in at least triplicate and subtracted from the average MTS signal for each condition. The % viability for the *ith* treatment condition was calculated according to

$$\%\;viability=100\times \frac{MTS_{i}-MTS_{background}}{MTS_{control}-MTS_{background}}$$

where MTS_i_, MTS_background_, and MTS_control_ are the MTS signals for the *i*th treatment condition, the average signal from the background wells, and the average MTS signal from the negative (untreated) control wells, respectively. To compare conditions where a single therapy had been used (*e.g*., MMT only), a one-tailed *t* test was used and was calculated using the data analysis package in Microsoft Excel. The null hypothesis (that the samples have no significant difference) was rejected for *p* < 0.05. For experiments combining multiple therapies (*e.g*., radiotherapy + MMT), 2 × 2 analysis of variance (ANOVA) was conducted using Welch statistics.^34^ Null hypotheses (3 in total) testing the effect of each therapy and of the interaction were used and rejected where *p* < 0.05. Using Welch statistics gives a more cautious estimate of the p-value than conventional ANOVA and accounts for samples with differing variances (ANOVA usually assumes equal variances).^35^

The cells were stained for iron using a Prussian Blue Iron StainKit and supplied by Abcam (containing potassium ferrocyanide solution, HCl, and Nuclear Fast Red). The medium was removed from the microplate wells, and the cells were rinsed with DPBS before fixing them in 3.7% methanol-stabilized aqueous formaldehyde solution (Sigma-Aldrich) for 15 min. The formaldehyde solution was removed, and the cells were rinsed with DPBS and then DI H_2_O. Equal volumes of potassium ferrocyanide solution and hydrochloric acid were mixed freshly before adding ∼100 μL to each well for 3 min to stain the iron blue. The cells were then rinsed with DI H_2_O before adding Nuclear Fast Red Solution for 5 min to stain the nuclei red. Finally, the wells were rinsed thoroughly in DI H_2_O and images were immediately obtained using a Nikon TS100 microscope equipped with a Canon DSLR camera for imaging. The percentage of iron associated with the cells was quantified by taking a ratio between the blue-stained and pink-stained regions in the images using a custom ImageJ script. The script runs a color deconvolution (Fast Red and Fast Blue) to separate the red and blue parts before quantifying the total area occupied by the red and blue by treating the areas as particles to analyze. The % iron associated with the cells used to assess the relative amount of iron in the image was calculated by normalizing the total iron area (blue) to the total cell area (sum of blue and pink areas) by the following equation

$$\%\;iron\;coverage=100\times \frac{A_{blue}}{A_{blue}+A_{pink}}$$

where *A*_blue_ and *A*_pink_ are the total areas of blue and pink in the image. The color deconvolution and particle analysis scripts are built into ImageJ.

### MMT and RT

The AMF applicator consisted of a Ferronato BH175HF-H1 single-axis Helmholtz coil (Serviciencia) (740 μT/A, inner diameter 16 cm, distance between coils 5.7 cm), supplied by a TS250 waveform amplifier (Accel Instruments) connected with an RS pro RSDG 805 function generator (RS components). Helmholtz coils generate uniform magnetic fields. The signal generator and amplifier are used to sinusoidally vary the current in the coil to change the size and direction of the magnetic field with time. The function generator was controlled to output a 1 Hz sine wave with a peak voltage (Vpp) 0.65 V into the amplifier set to DC mode (which is for operations below 50 Hz) an input impedance of 50 Ω, and a 20 dB gain. Current and voltage measurements were taken by using an RS Pro IDS6072 AU digital oscilloscope. Magnetic field measurements were taken using a Hirst Magnetics GM07 G Meter with an axial probe measuring the positive peak of the time-varying AC magnetic field (which was determined to be 0.76 mT at 0.65 Vpp at 1 Hz). For applying MMT, the microplate was located centrally within the coil on a plastic pillar, and the field was applied at 0.76 mT, 1 Hz for 1 h. MMT was applied post seeding at 1 h, 24 h, and 48 h. RT was carried out using a γ Service GSR D1 equipped with a high activity (∼200TBq) Cs-137 source emitting γ radiation at 0.6617 MeV. The microplates were placed in the chamber at room temperature and irradiated at a constant dose rate of 0.019 Gy/s. The dose rates were determined to be uniform across a large (30 cm) area of the instrument. For applying RT, the microplate was placed in the γ Service GSR D1 and exposed for an amount of time to provide a dose between 0 and 20 Gy. RT was applied between 1 and 4 h post seeding. RT (if applied in addition to MMT) was performed after the 1 h MMT.

### S-SNOM and AFM

Samples were prepared by growing cells directly on 5 mm × 5 mm poly-l-lysine-coated boron-doped ultraflat silicon wafer chips (NanoAndMore), prepared by incubating ultraviolet (UV)-sterilized wafers in 0.01% poly-l-lysine solution (Sigma-Aldrich, U.K.) before allowing the wafers to air-dry under a laminar flow biosafety hood. The wafers were placed in 12-well microplates prior to cell seeding. Following cell growth and treatment, the samples were rinsed in 0.1 M 4-(2-hydroxyethyl)-1-piperazineethanesulfonic acid (HEPES) pH 7.2, and fixed using 2.5% formaldehyde solution in 0.1 M HEPES at room temperature before rinsing the samples 3 times in 0.1 M HEPES. The samples were then dehydrated in a graded series of ethanol (5 min each 12.5, 25, 50, 70, 95% (twice), 100%) before replacing with 1:1 hexamethyldisilazane (HMDS)/ethanol (10 min) and finally HMDS for 10 min (twice). The samples were then allowed to air-dry at room temperature. AFM and s-SNOM images were acquired simultaneously by using a neaSNOMsystem (NeaSpec, Germany) with a quantum cascade laser (Daylight Solutions). Commercially available probes (Arrow NCPt, NanoWorld, Switzerland) with a tapping frequency of 285 kHz were used. AFM and s-SNOM images were processed using Gwyddion (v. 2.55) to remove line noise. A built-in microscope working in reflection mode using a white-light-emitting diode (LED) for illumination and a CMOS camera was used to record lower-magnification images.

## Supplementary Material



## Notes

Additional experimental results showing characterization of the AuFe NWs; example nanowire deposits on PCTE templates; and effect of the AuFe NWs on the viability of U87 cells (MTS assays, dose response) (PDF)

## Abbreviations

AC: alternating current
AFM: atomic force microscopy
AMF: alternating magnetic field
AuFe: NW(s) Gold–iron nanowire(s)
BF-TEM: bright-field transmission electron microscopy
DCM: dichloromethane
DMSO: dimethyl sulfoxide
EDTA: ethylenediaminetetraacetic acid
EDX: energy-dispersive X-ray spectroscopy
FBS: fetal bovine serum
FTIR: Fourier transform infrared spectroscopy
GBM: Glioblastoma multiforme
HAADF: high-angle annular dark-field scanning transmission electron microscopy
ICP-OES: inductively coupled plasma optical emission spectroscopy
IP: in plane
LSV: linear sweep voltogram
PEG: poly­(ethylene glycol)
MMT: magneto-mechanical therapy
OOP: out of plane
PCTE: polycarbonate track-etched
PPMS: physical property measurement system
RT: radiotherapy
ROS: reactive oxygen species
SERS: surface-enhanced Raman spectroscopy
SEM: scanning electron microscopy
s-SNOM: scattering-type scanning near-field optical microscopy
STEM: scanning transmission electron microscopy
TEM: transmission electron microscopy
TGA: thermogravimetric analysis
VSM: vibrating sample magnetometry
XRD: X-ray diffraction
