# Supplementary material for: Remote Control of Gold–Iron Nanowires Using Low-Frequency 1 Hz Magneto-Mechanical Therapy and Cesium 137 0.662 MeV Radiotherapy for Treatment of Glioblastoma Multiforme
Source: ACS Appl Mater Interfaces. 2025 May 28;17(23):33569–80. doi: 10.1021/acsami.5c05004 (PMC12163923; doi:10.1021/acsami.5c05004)
Supplement: Supplementary file 1 [file am5c05004_si_001.pdf]

Supporting Information

Remote control of gold-iron nanowires using low frequency 1Hz Magneto-mechanical Therapy and Cesium 137 0.662 MeV Radiotherapy for Treatment of Glioblastoma Multiforme

Jonathan Taylor[1], George Greaves[2], Chris Clement Phillips[2], Matthew Williams[3], Mary P. Ryan[1], Alexandra E. Porter[1]*

[1]Department of Materials and London Centre for Nanotechnology, Imperial College, London SW7 2AZ, UK.

[2] Department of Physics, Imperial College London , SW72AZ, UK.

[3] Imperial College Healthcare NHS Trust, Charing Cross Hospital, Fulham Palace Rd, London W6 8RF, UK.

*Corresponding author: a.porter@imperial.ac.uk

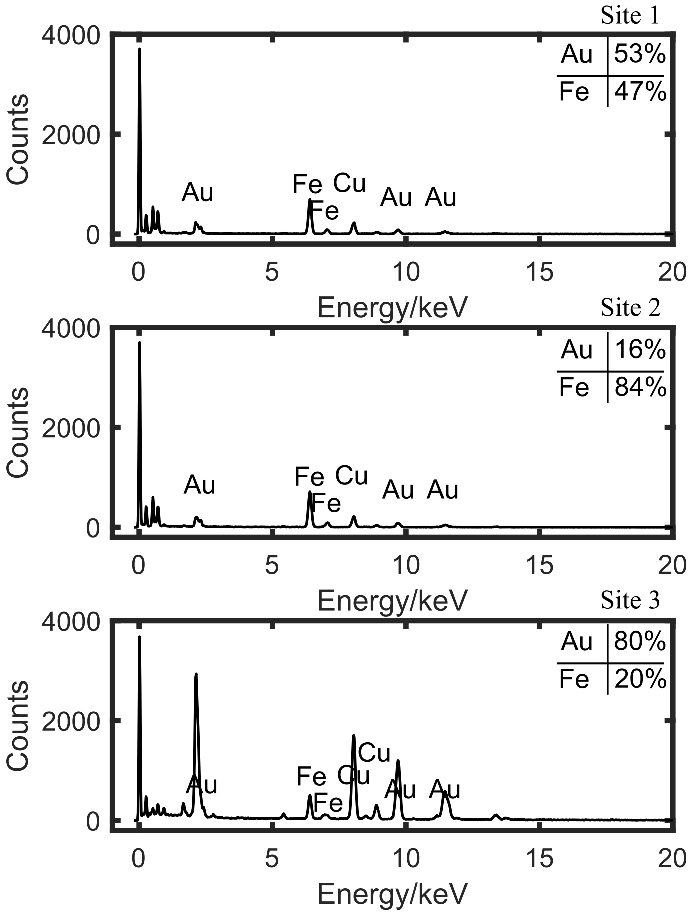

Figure S1. EDX spectra taken at sites 1 (top panel), 2 (middle panel), and 3 (bottom panel) marked in Figure 2D.2. Tables show quantification of gold and iron percentage by number.

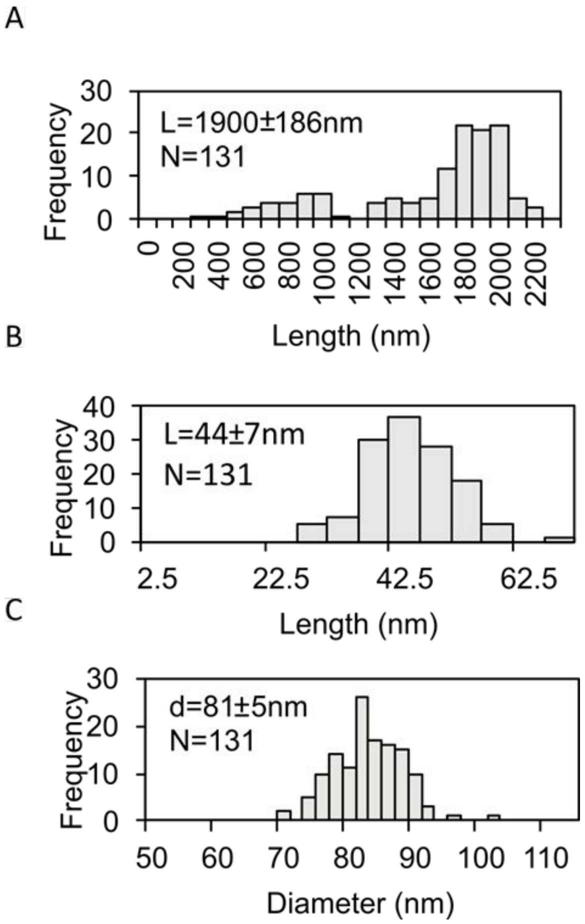

Figure S2: Size distribution of AuFe NWs derived from BF-TEM data (e.g. as in Figure 2D.1) showing (A) total length, (B) length of gold section, and (C) diameter.

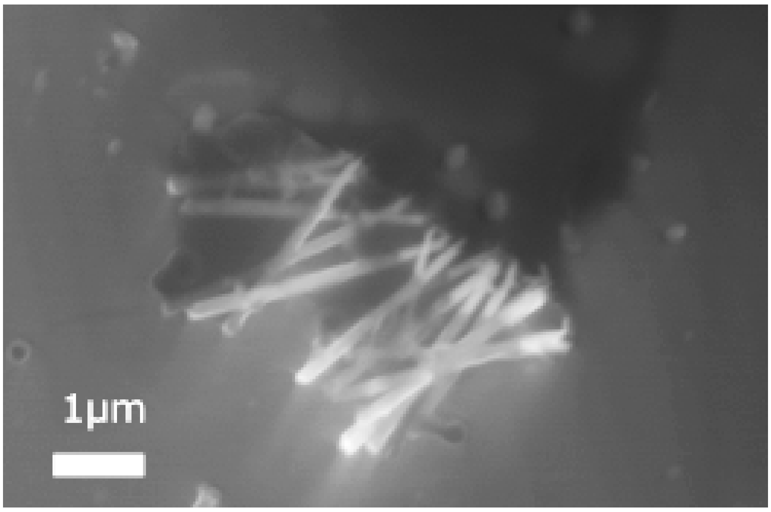

Figure S3: SEM micrograph of AuFe NWs.

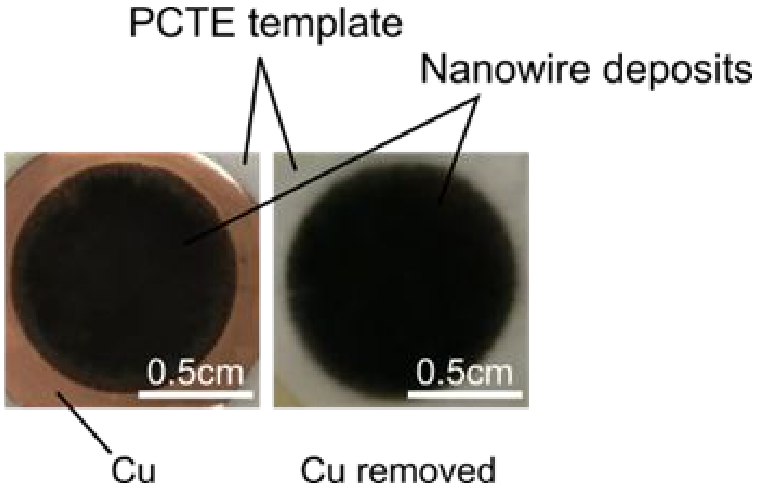

Figure S4: Photograph showing example nanowire deposits on PCTE templates. Left image shows nanowire deposits on PCTE template with copper electrode. Right image shows the same as the left, but with the copper removed by chemical etching.

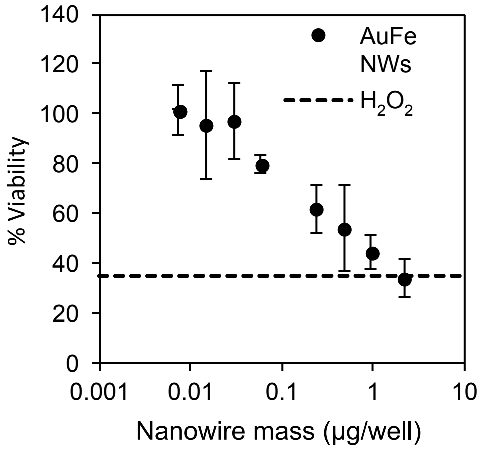

Figure S5. MTS assays showing cell viability 48 hours after seeing of U87 cells treated with various doses of AuFe nanowires by total nanowire mass (n= 3).
